# Tumor Treating Fields (TTFields) Therapy in Unresectable Pleural Mesothelioma: Overview of Efficacy, Safety, and Future Outlook

**DOI:** 10.1007/s11864-025-01320-w

**Published:** 2025-04-23

**Authors:** Giovanni Luca Ceresoli, Letizia Gianoncelli

**Affiliations:** 1 Medical Oncology Unit, Cliniche Humanitas Gavazzeni, Via Mauro Gavazzeni, 21, Bergamo, Italy; 2 Medical Oncology Unit, ASST Santi Paolo E Carlo, Ospedale San Paolo, Via Antonio Di Rudinì, 8, 20124 Milan, Italy

**Keywords:** Mechanism of action, Medical device usage, NovoTTF- 100L, NovoTTF- 200 T, Pleural mesothelioma, Tumor Treating Fields

## Abstract

Pleural mesothelioma is an incurable cancer with unmet diagnostic and therapeutic needs. Due to its pattern of local spread, few patients are candidates for multimodality treatment and thus most patients only receive systemic therapy. Chemotherapy (pemetrexed plus platinum) was standard of care until the recent addition of immunotherapy (nivolumab plus ipilimumab, or pembrolizumab plus chemotherapy) as further first-line option. Physicians treating pleural mesothelioma should be aware of another option with Tumor Treating Fields (TTFields) therapy, a locoregionally-applied therapy utilizing electric fields generated by a portable medical device, and delivered to the tumor by skin-placed arrays. TTFields therapy delivered to the thorax using the NovoTTF- 100L device concomitant with pemetrexed and platinum agent is approved for unresectable pleural mesothelioma in the US, and received Conformité Européenne certification in Europe, based on results from the phase 2 STELLAR study (EF- 23; NCT02397928), where TTFields-related toxicity was limited to mild-to-moderate reversible skin reactions. Overall survival in the STELLAR study with TTFields therapy was 18.2 months, with further post-hoc analysis showing extended survival in patients with epithelioid histology. Within the evolving landscape of systemic treatments, TTFields therapy represents a novel and clinically versatile therapeutic option in the battle against pleural mesothelioma without introducing additional toxicities other than mild-to-moderate skin irritation. While promising, additional research is needed to optimize clinical application of TTFields therapy in patients with pleural mesothelioma, such as identifying the molecular determinants of therapy efficacy, and further investigation into the safe and effective delivery of TTFields therapy together with systemic agents, including immunotherapies.

## Introduction

Pleural mesothelioma is an asbestos-related, highly aggressive tumor whose global incidence continues to rise [1–3]. Few patients with unresectable disease are eligible for multimodal therapy that includes surgery, and most only receive systemic therapy [4].

The first systemic therapeutic option to demonstrate a survival benefit in patients with unresectable pleural mesothelioma was the combination of cisplatin and pemetrexed, which was established as standard of care in 2004 following the results of the phase 3 EMPHACIS trial [5, 6]. For elderly patients, or patients unfit to receive this doublet, schedules with carboplatin were implemented, with similar outcomes but better toxicity profiles [7, 8]. Otherwise, despite several phase 2 and 3 trials, first-line treatment remained unchanged for 15 years. The phase 3 MAPS trial showed addition of bevacizumab to pemetrexed plus cisplatin chemotherapy to significantly improve overall survival (OS) in pleural mesothelioma (median OS 18.8 months vs 16.1 months without bevacizumab; *p* = 0.0167), with a manageable toxicity profile [9], and the combination was subsequently included as a recommended first-line treatment option across treatment guidelines [4, 10]. The next positive phase 3 study was CheckMate 743 (NCT02899299), in which first-line immune checkpoint inhibitors [ICIs] (a combination of nivolumab and ipilimumab) demonstrated a significant survival benefit over standard chemotherapy (median OS 18.1 months vs 14.1 months, respectively; *p* = 0.002), leading to approval of this regimen by the US Food and Drug Administration (FDA) in 2020 and the European Medicines Agency (EMA) in 2021 [11–13]. Most recently, IND.227 (NCT02784171), also known as KEYNOTE- 483, demonstrated a significant survival benefit over standard chemotherapy with another ICI, pembrolizumab, in combination with pemetrexed and platinum chemotherapy, leading to subsequent approval by the FDA and a recent approval by the EMA for non-epithelioid patients only in first line treatment [14, 15].

There are no approved second-line therapies for patients with disease progression on or after first-line treatment [4]. In clinical practice, patients are usually treated with single-agent chemotherapy (vinorelbine, gemcitabine, or pemetrexed rechallenge) [16–18]. Other novel chemotherapeutic agents, such as trabectedin [19] and lurbinectedin [20], have shown limited activity. Moreover, although early phase, single-arm trials were encouraging, randomized phase 3 studies of ICIs versus single-agent chemotherapy, or placebo, have shown inconsistent results [21–24]. Targeted therapies, widely investigated in recent years, have generally shown no significant improvement in patient survival [25, 26] although recently, a promising survival improvement was reported in a phase 2 study with the combination of gemcitabine and the anti–vascular endothelial growth factor- 2 agent, ramucirumab [27].

Tumor Treating Fields (TTFields) are electric fields that disrupt processes critical for cancer cell survival and tumor progression. TTFields therapy is delivered locoregionally and noninvasively to the tumor site by a portable medical device via two pairs of arrays placed on the patient’s skin around the tumor site [28]. As of 2024, TTFields therapy is FDA-approved in four indications. The first approval was granted in 2011 for the treatment of recurrent glioblastoma, following the results of the pivotal EF- 11 study (NCT00379470) that demonstrated similar efficacy outcomes compared to physician’s best choice of chemotherapy, but with fewer severe adverse events and better quality of life [29, 30]. In 2015, the FDA granted additional approval for TTFields therapy in newly diagnosed glioblastoma concurrent with maintenance temozolomide after standard chemo-radiation, based on a significant improvement in OS versus temozolomide alone in the pivotal, randomized EF- 14 study (NCT00916409) [29, 31, 32]. In 2019, based on data from the phase 2 STELLAR study [33], the FDA and EMA approved TTFields therapy concomitant with pemetrexed and platinum-based chemotherapy for unresectable locally advanced or metastatic pleural mesothelioma [34, 35]. Most recently, the FDA approved TTFields therapy applied concomitant with a PD- 1/PD-L1 inhibitor or docetaxel in patients with metastatic non-small cell lung cancer (NSCLC) that had progressed on or after a platinum-based therapy [36, 37]. TTFields therapy given concurrently with standard of care therapies is also in clinical development as a potential approach for several other solid tumor indications [38–41].

Here we review the TTFields mechanism of action and preclinical evidence for efficacy in pleural mesothelioma, including data showing that mesothelioma cell lines are among the most sensitive to TTFields treatment [42–44]. We then consider the application of TTFields therapy to the thorax in the clinical management of advanced pleural mesothelioma, including safety and efficacy of TTFields therapy concomitant with standard chemotherapy. Furthermore, we will highlight future perspectives on TTFields therapy in clinical practice and research for patients with pleural mesothelioma.

### TTFields Mechanism of Action

The application of TTFields-generated electric fields to cells applies forces on charged and polarizable molecules and objects. Since electrostatic forces are involved in a variety of physiological and pathological processes, TTFields affect cancer cells via multiple mechanisms of action including disruption of mitosis resulting in downstream enhancement of antitumor immunity, interference with cellular motility and migration, downregulation of the DNA damage response, and increased cancer cell membrane permeability [44–50]. TTFields selectively affect tumors, while sparing cells in surrounding healthy tissues, due to the distinct characteristics of cancer cells, including proliferation rate, morphology, and electrical properties [28, 51–57]. Furthermore, the effects of TTFields are frequency-specific and can be empirically tailored to each specific tumor cell-type, and correlate with treatment duration and field intensity [45, 58].

During metaphase, TTFields disrupt the alignment of highly polarized tubulin subunits, and thus interfere with formation of the mitotic spindle [42, 45, 58]. The mitotic process is further perturbed by the action of TTFields on the localization of septins, key proteins involved in cellular division [59]. During telophase, the transition of cancer cell to an hourglass shape causes a non-uniform electric field, resulting in movement of charges and dipoles (i.e. intracellular polarized macromolecules and organelles) toward the area of highest TTFields intensity in a process called dielectrophoresis [60]. Through movement of polar biomolecules close to the mitotic furrow, TTFields disrupt cell integrity at the cleavage plane, leading to defective telophase/cytokinesis [59, 60]. Ultimately, the failure of mitosis may lead to mitotic cell death or, more commonly, to mitosis completion while forming abnormal progeny [42]. Such progeny may undergo permanent arrest or succumb to internal stress and die during interphase [42].

The stress experienced by abnormal progeny cells following TTFields application induces the activation of autophagy [47, 61–65], an intracellular degradative process that occurs under stressful conditions such as organelle damage, the presence of abnormal proteins, or nutrient deprivation [66]. The process of autophagy includes autophagosome formation, maturation, fusion with lysosomes, and lysosomal degradation of cytoplasmic constituents. While the induction of autophagy may lead to reduced sensitivity to the stress induced by TTFields and reduced sensitivity to TTFields as a treatment [61], it may also help enhance a TTFields-triggered immune response [47].

The first evidence that TTFields therapy itself may activate an antitumor immune response came from an early study using a rabbit model of metastatic cancer, which reported an abscopal effect on lung metastases following locoregional TTFields treatment of a squamous cell carcinoma implanted within the kidney capsule. A significant increase of T-cell infiltration in the lungs of treated animals was observed, suggesting that the effect was mediated by the immune system [67]. In vitro data from a study investigating the mechanistic basis of the effect identified that several cancer cell lines treated with TTFields had increased immunogenic cell death (ICD) markers, such as elevated phosphorylation of eukaryotic translation initiation factor 2α (eIF2α) with subsequent calreticulin translocation to the cell surface, release of high-mobility group box 1 protein (HMGB1), and autophagy-dependent ATP release [47]. Correspondingly, indicators of ICD induction (increased circulating levels of HMGB1 and intratumoral levels of phosphorylated eIF2α) were shown to be elevated by TTFields treatment in a mouse model of lung cancer, thus confirming the in vitro observations [68]. There is also evidence that TTFields induces adaptive immunity, via stimulating the production of immune-stimulating proinflammatory cytokines by a mechanism involving the cGAS/STING axis and activation of AIM2 inflammasomes. The effect was seen in in vitro and *in vivo* models of glioblastoma, and transcriptional profiling also detected robust activation of adaptive immunity in patients with glioblastoma following treatment with TTFields therapy [69]. The effect of giving TTFields treatment concomitantly with immunotherapy has also been studied in mouse models of lung cancer. Voloshin et al. demonstrated that concurrent administration of TTFields with anti–PD- 1 therapy significantly decreased tumor volume, compared with either modality alone [47]. Barsheshet et al. showed similar decreases in tumor volume when TTFields was administered concomitantly with anti–PD- 1/anti–CTLA- 4, or an anti–PD-L1 therapy [68]. Both studies reported a higher frequency of tumor-infiltrating lymphocytes in orthotopic lung models (i.e. implantation of cancer cells of the same origin as the organ or tissue in which they are transplanted; in this case murine lung cancer cells transplanted into the lung) following concurrent administration of TTFields treatment with immunotherapy, compared with TTFields or immunotherapy alone. Accordingly, there was elevated expression of PD-L1 in infiltrating macrophages and dendritic cells alongside increased production of interferon-γ in cytotoxic T cells isolated from these tumors [47, 68]. Considered overall, the benefit of concomitant application of TTFields with immunotherapy provides further evidence for the immune-activating role of TTFields.

It is increasingly clear that other mechanisms of action also contribute to the therapeutic effects of TTFields, some of which may be particularly important when TTFields therapy is used concomitant with certain systemic therapies, including chemotherapies [52, 70, 71]. Studies in several cancer cell types have demonstrated increased DNA damage following application of TTFields, and they have been suggested to interfere with the efficiency of DNA repair, downregulating Fanconi anemia (FA)-BRCA DNA repair pathway genes [44, 46, 49, 72]. Importantly, the effect of TTFields on DNA damage repair mechanisms, i.e. the induction of a ‘BRCAness’ state in the cells, has been shown to increase the efficacy of other DNA damage-inducing cancer modalities such as chemotherapy and radiotherapy [44, 46, 49, 58, 72].

TTFields have also been shown to reversibly alter cellular membrane structure in cancer cell models, thus potentially increasing permeability to chemotherapeutics [50]. This may contribute to the beneficial effects seen when co-administering TTFields with chemotherapy in preclinical and clinical studies. The effect was confirmed by electron microscopy that showed an increase in the number and size of fenestrations in the membrane of treated cancer cells [50]. Importantly, TTFields did not cause membrane holes in normal human fibroblasts, thus suggesting this phenomenon might be specific to neoplastic cells [50], possibly due to their altered physical–chemical properties.

Similar to the impact on microtubules during mitosis, TTFields effects on the cytoskeleton lead to reduced cancer cell motility and invasion [64, 65, 73]. Changes in microtubule organization, leading to broad effects on cytoskeletal dynamics and cancer cell motility, have been reported following *in vitro* application of TTFields to cancer cells [48]. The alterations in microtubule organization led to increased activation of guanine nucleotide exchange factor-H1 (GEF-H1), triggering the Ras homolog family member A (Rho A)/Rho-associated coiled-coil kinase (ROCK) signaling cascade (a key regulator of the cytoskeleton), and ultimately resulting in the formation of focal adhesions and actin reorganization [48]. In addition, TTFields inhibited glioblastoma cell invasion and migration *in vitro*, via dysregulation of epithelial-to-mesenchymal transition-related proteins, alongside down-regulation of nuclear factor (NF)-κB-, mitogen-activated protein kinase (MAPK) and phosphatidylinositol 3-kinase (PI3 K)/Akt-dependent pathways [73].

### TTFields in Preclinical Models of Pleural Mesothelioma

Consistent with other tumor types, the anticancer effects of TTFields treatment on pleural mesothelioma cell lines was dependent on its frequency, intensity, and duration [44, 74]. The optimal frequency that produces the greatest percent reduction in viable mesothelioma cell numbers has been established at 150 kHz [44].

Recent preclinical work has investigated why different histological subtypes of mesothelioma are associated with different clinical outcomes. Mannarino, et al. found that epithelioid CD473 cells were more sensitive to TTFields treatment than sarcomatoid CD60 cells [43]. TTFields treatment differentially affected cell cycle progression in the different subtypes, with a G2/M-phase block in the most sensitive cells, and a milder general cell cycle delay in less sensitive cells. The transcriptional changes induced by TTFields treatment were also greater in CD473 than CD60 cells. Additionally, the data suggested that the antitumor effects of TTFields in mesothelioma were related to the biological background of the cancer cells, and not a simple non-specific cellular reaction to a physical stimulus [43].

Preclinical studies investigating different types of tumor cell type have found that mesothelioma cell lines are particularly sensitive to TTFields in terms of cell viability and clonogenicity [42]. Their sensitivity may relate to the increased formation of DNA double strand breaks, increased expression of DNA damage-induced cell cycle arrest proteins, and reduced expression of FA-BRCA pathway proteins shown in mesothelioma cells under TTFields treatment [44]. The same study also showed that TTFields treatment applied to mesothelioma cell lines concomitant with cisplatin and pemetrexed induced a shift to the left of the dose–response curve of both agents, indicative of increased activity compared to chemotherapy alone [44]. The dose–response shift also varied by the type of chemotherapy, with an overall additive response for TTFields treatment plus pemetrexed, while synergism was demonstrated with cisplatin. The enhanced effect with cisplatin is potentially because of the impact of TTFields on the FA-BRCA pathway that, in turn, is required to repair damage caused by DNA cross-linking platinum-based agents like cisplatin [44]. These data also again highlight that the anticancer effects of TTFields treatment arise via specific effects on cancer cell biology.

In vivo animal models also support that TTFields treatment with chemotherapy has activity in pleural mesothelioma. TTFields treatment (1.2 V/cm, 150 kHz for 8 days) concomitant with pemetrexed/cisplatin chemotherapy significantly reduced tumor volume (compared to no treatment) in rats injected with interleukin- 45 mesothelioma cells into the pleural cavity, while pemetrexed/cisplatin chemotherapy alone did not [44]. Similarly, 7 days of TTFields treatment given concomitant with pemetrexed/cisplatin to mice inoculated with RN5 mesothelioma cells resulted in significantly smaller tumors than the chemotherapy doublet alone [44]. Importantly for its clinical use, there were no pathological findings in the major internal organs of healthy rats after TTFields (1–2.5 V/cm, 150 kHz) was applied continuously to the torso for 2 weeks [51].

### TTFields Therapy with the NovoTTF- 200 T System in Clinical Practice

**Fig. 1:**
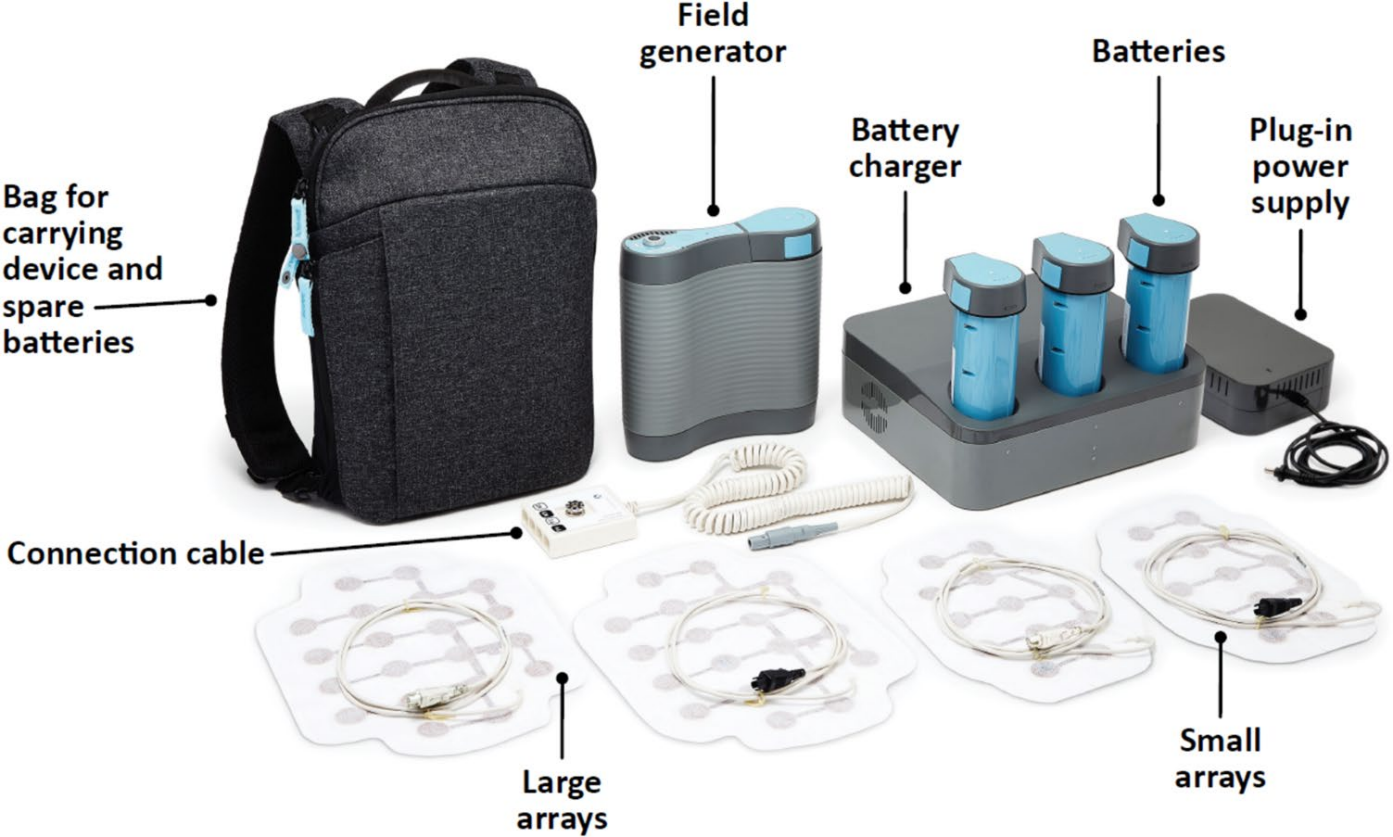
The NovoTTF™− 200 T system is comprised of an electric field generator and new generation arrays. It is the first device indicated to be used in conjunction with standard chemotherapy to treat patients with unresectable pleural mesothelioma, US Food and Drug Administration approval date: May 23, 2019 [34].

The first generation NovoTTF™− 100L system (Novocure Ltd) received FDA approval for unresectable pleural mesothelioma in 2019 [34]. The system consists of two main components: a portable electric field generator (the NovoTTF device) and insulated arrays. Additional components are a power supply, rechargeable batteries, a battery charger, connection cable, and a carrying backpack (Fig. 1) [75, 76]. The medical device was subsequently updated to a second generation model (NovoTTF- 200 T, also known as Optune Lua™ [Novocure Ltd]) that is smaller and lighter than its predecessor (1.2 kg vs 2.7 kg) and uses ITE arrays, but has the same essential components [75, 77].

**Fig. 2:**
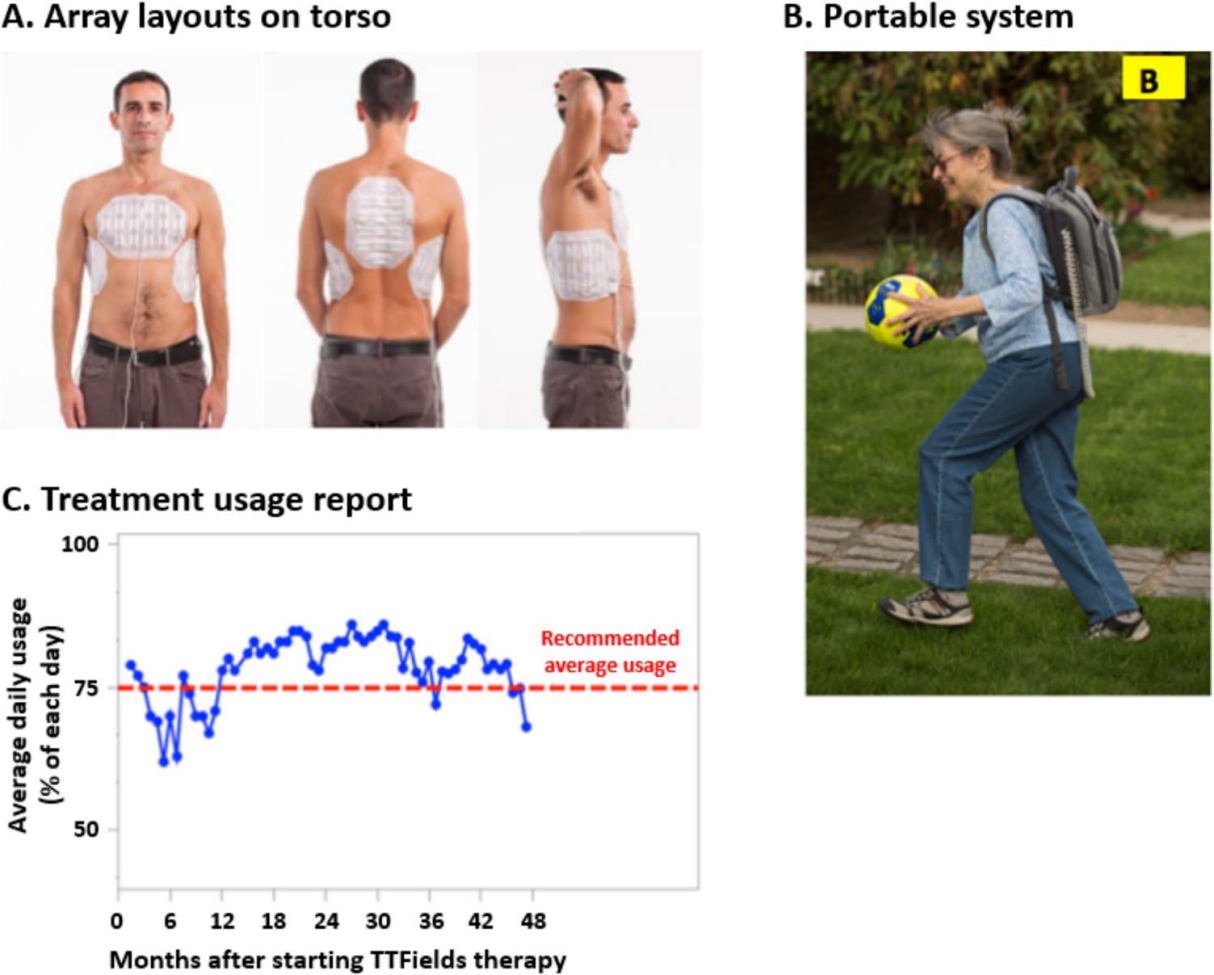
**A** The arrays are placed on opposite sides of the torso and **B** can be arranged under clothes. **C** Daily use is recorded to allow monthly reviewing. Photos are of models and not patients**,** *TTFields* Tumor Treating Fields.

Patients with unresectable pleural mesothelioma receive TTFields therapy with output parameters set to 150 kHz, and two sequential, perpendicular field directions, with a maximal intensity of 1414 mA root mean square power [75]. Each array is composed of electrodes connected in a flexible circuit board and embedded in an adhesive patch, with two sizes of arrays available for clinical use: a large array with 20 electrodes (ceramic discs) and a small array with 13 electrodes [29, 35] (Fig. 1). The arrays are placed on the patient’s skin above and surrounding the tumor. The array size and placement are selected to maximize the electric field intensities around the tumor, using clinical guidelines per tumor location, and also depending on patient gender and body size; they may be placed on opposite sides of the body, front and back, or one pair of arrays may be placed front and back, and the other pair on the sides of the torso (Fig. 2A) [35].

TTFields therapy is administered continuously with a recommended average duration of at least 18 h per day (75% per day) [35]. The medical device is preset with treatment parameters to avoid the need for any electrical output adjustments by the patient or the treating physician. Patients and their caregivers only need to learn to change and recharge depleted batteries, and to connect to an external power supply (for example, overnight) [35], and Novocure Device Support Specialists provide assistance with technical training and education [78]. The device is portable (in an over-the-shoulder bag or backpack [75, 76]) so that patients experience minimal impact in their daily living; the cables connecting the field generator to the arrays can be arranged under clothes (Fig. 2B). Of note, electric fields of the frequency and intensity used in TTFields therapy pose no danger to anyone in close physical proximity to the patient [78].

Ideally, a patient receiving TTFields therapy should have at least one caregiver who can help with removing and accurately reapplying the arrays, as well as assist with management of adverse events (for further information see section on skin care) [78]. Patient and caregiver education and training in advance of starting TTFields therapy are therefore an essential part of treatment. Both should have a basic understanding of the components of the NovoTTF- 200 T system, and should be informed of the importance of achieving the recommended daily usage [78] (discussed further below). To aid the latter, the medical device outputs generate a monthly report that allows the patient and health care team to track daily usage time (Fig. 2C) [78]. This is a valuable tool to monitor patient time on therapy, and to provide counseling and coaching for optimal treatment use.

The benefits and risks of TTFields therapy should be discussed with patients and caregivers before starting any treatment; a careful evaluation of possible exclusion criteria is mandatory. Patients with implanted electronic medical devices such as cardiac pacemakers and defibrillators, or who require supplemental oxygen, should not receive TTFields therapy [75]. In addition, TTFields therapy may not be suitable for patients who live alone and have no caregiver, or who have had difficulties using a localized therapy.

### The STELLAR Study

The STELLAR study, a prospective, international, phase 2, single-arm study, evaluated the activity and safety of TTFields therapy delivered to the thorax concomitant with standard chemotherapy in patients with untreated, unresectable pleural mesothelioma [33]. Patients received continuous TTFields therapy at a frequency of 150 kHz with the NovoTTF- 100L system, for a planned duration of at least 18 h per day, concomitantly with up to six cycles of standard chemotherapy with pemetrexed and platinum (investigator’s choice of cisplatin or carboplatin). Patients who remained progression-free after completing TTFields therapy concomitant with chemotherapy, continued with TTFields therapy alone as maintenance until disease progression, unacceptable toxicity, or the patient or physician decided to discontinue for any reason. The primary endpoint of the study was OS, and secondary endpoints were progression-free survival (PFS), radiological response rate according to modified Response Evaluation Criteria in Solid Tumours criteria [79], and safety.

**Fig. 3:**
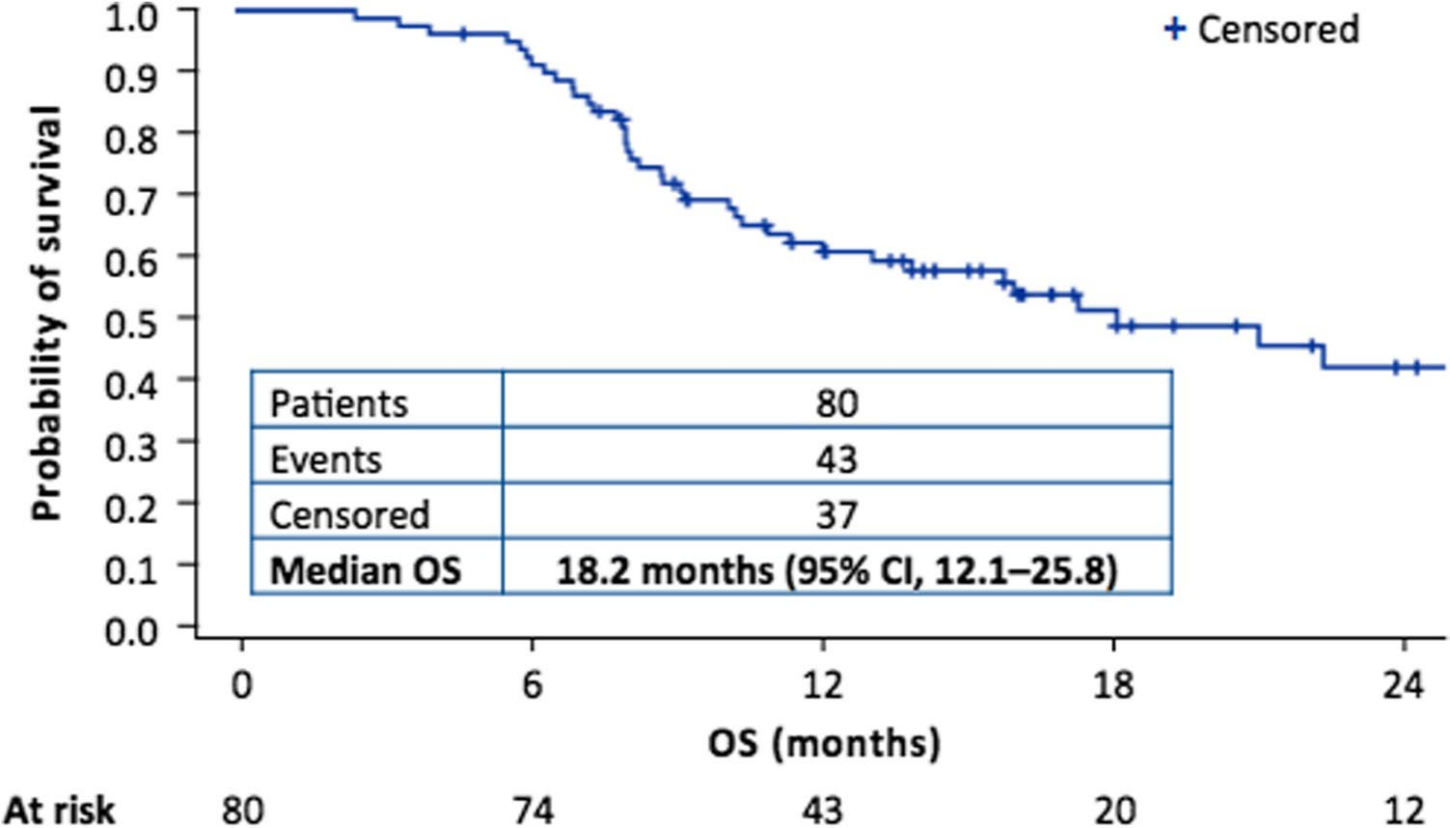
Kaplan–Meier curve of overall survival (OS) in the STELLAR study, Adapted from Ceresoli GL et al. Lancet Oncol. 2019;20:1702–9 [33].

From February 2015 to March 2017, 80 patients were enrolled at 12 European sites [33, 80]. Median age was 67 years, with a broad range of 27–78 years (Table 1). Fifty-three patients (66%) had disease with epithelioid histology, 21 (26%) had a non-epithelioid (sarcomatoid/biphasic) histological subtype, and six (8%) had disease of unknown subtype. Patients received a median of eight 3-week cycles of TTFields therapy, and a median of six chemotherapy cycles. Fifty patients (63%) received the carboplatin/pemetrexed chemotherapy combination. Median OS was 18.2 months, with 62% of patients alive at 1 year, and a 2-year OS rate of 42% (Fig. 3). Patients with epithelioid tumors survived longer, with a median OS of 21.2 months versus 12.1 months for those with non-epithelioid tumors. Follow-up was performed every 3 weeks, and a radiological assessment (at study sites) was performed every 6 weeks until disease progression. Median PFS was 7.6 months in the overall study population (8.3 and 6.5 months in patients with epithelioid and non-epithelioid histology, respectively). Among 72 patients with an evaluable response per modified Response Evaluation Criteria in Solid Tumours criteria, partial responses (PRs) were observed in 29 patients (40%), and clinical benefit (PR or stable disease [SD]) was achieved in 70 patients (97%). The median duration of response was 5.7 months. After progression, 44 patients (56%) received post-study therapy (primarily second-line chemotherapy or pemetrexed re-challenge); progressive disease or short-lived SD was the best response to post-study therapy in most patients. The addition of TTFields therapy to standard chemotherapy was not associated with any additional systemic adverse events than would be expected with chemotherapy alone. The only toxicity related to the TTFields device was skin irritation underneath the arrays. Grade 1–2 skin reactions were observed in 53 patients (66%); only four patients (5%) had a grade 3 skin adverse event, leading to temporary or permanent interruption of the treatment. In the majority of cases, skin irritation was resolved with regular shifting of the arrays applied to the skin, topical corticosteroids, or short treatment breaks.

**Table 1:** STELLAR study: patient characteristics

Baseline characteristics and treatments (*N* = 80)	
Age, median years (range)	67 (27–78)
Sex, n (%)	
Male	67 (84)
Female	13 (16)
ECOG performance status	
0	45 (56)
1	35 (44)
Histology, n (%)	
Epithelioid	53 (66)
Sarcomatoid/biphasic	21 (26)
Unspecified	6 (8)
Chemotherapy cycles, median (range)	6 (1–6)
TTFields cycles, median (range)	8 (2–41)
Patients treated with carboplatin, n (%)	50 (63)

Data are compiled from references [33, 75], *ECOG* Eastern Cooperative Oncology Group; *TTFields* Tumor Treating Fields

The STELLAR study had the limitations inherent to a single-arm phase 2 study, including a potential sampling bias for a heterogeneous cancer like pleural mesothelioma. However, the sample size was relatively large, and patient baseline characteristics, including Eastern Cooperative Oncology Group performance status, were comparable to other first-line pleural mesothelioma studies, such as the MAPS (NCT00651456) and LUME-Meso trials (NCT01907100) [9, 81]. It was also unlikely that post-study treatments influenced survival outcomes.

### Increasing TTFields Medical Device Usage

A case study of two patients who participated in the STELLAR study described notable long-term progression free survival of 16 months and 8 years. Both of these patients achieved usage of 75% or 18 h per day [82]. Although there are minimal data available about the relationship between the duration of TTFields therapy use and outcomes in patients with pleural mesothelioma, use of the NovoTTF- 100 A (Optune) medical device has been shown to correlate with the efficacy of TTFields therapy in patients with glioblastoma. Usage time of TTFields therapy was an independent predictor of improved OS in the EF- 11 study of patients with recurrent glioblastoma, with a median OS of 7.7 months in patients with at least 18 h per day usage, versus 4.5 months in patients who used the device < 18 h per day (p = 0.04) [83]. A similar relationship between device use and outcomes was seen in patients with newly diagnosed glioblastoma treated in the EF- 14 study. A threshold value of 50% compliance with TTFields was needed for improved PFS (HR 0.70, 95% CI 0.47–1.05) and OS (HR 0.67, 95% CI 0.45–0.99), with outcomes further improving as usage increased. In particular, patients with usage greater than 90% showed the highest median OS (25 months) and 5-year survival rate (29%) [84].

In the STELLAR study [33], patients were required to receive continuous TTFields therapy by the NovoTTF- 100L system for at least 18 h per day. The device automatically reported daily use for review by the treating team, patient, and caregivers. Over the first 3 months of TTFields therapy, median use was 16.3 h per day for the overall population of patients [33], slightly lower than the recommendation. This includes the four patients who temporarily paused therapy due to device-related skin toxicity, 38 patients (47%) who had a temporary therapy break of 3 or more days (throughout the entire treatment period) for reasons unrelated to toxicity, and four patients (5%) who permanently discontinued TTFields therapy before having progressive disease [33]. Real world data on five patients with unresectable pleural mesothelioma treated at a single center in Miami, Florida (USA) noted their usage from the first 3 months of TTFields therapy (concomitant with pemetrexed and platinum, and using the original NovoTTF- 100L system) was for a median of 12.5 h per day [85]. The investigators concluded that a number of factors probably contributed to the lower than recommended usage, including that they resided in a hot, humid climate, and that four patients had recurrent pleural mesothelioma and thus represented a particularly frail patient population. Additionally, all five patients experienced skin adverse events – two at grade 1 and three of grade 2 – that required treatment interruptions [85].

These findings suggest it may be important to identify approaches to improve device usage in patients with pleural mesothelioma. Strategies aimed at reducing skin reactions are essential, given that dermatological adverse events negatively affected treatment continuity in STELLAR [33], and that itchy skin was the only quality of life (QoL) measure worsened by TTFields therapy in patients with recurrent glioblastoma [86]. Additionally, the second generation TTFields medical device is smaller and less than half the weight of the original NovoTTF- 100L system (2.7 lbs [1.2 kg] vs 6 lbs [2.7 kg], respectively; Table 2) [78].As a result, the second generation device may be less challenging for frail or unfit patients with pleural mesothelioma to use. The second-generation system also is easier to use: patients and caregivers are able to track battery levels more easily and the device, also includes improved electronic components, circuit boards, and digital signaling technology [78].

**Table 2:** First- and second-generation TTFields medical devices

	First-generation (NovoTTF- 100 system)	Second-generation (NovoTTF- 200 system)
Total weight (device plus battery)	2.7 kg	1.2 kg
TTFields generator (device) weight	0.9 kg	0.7 kg
Battery weight	1.8 kg	0.5 kg
Size	Device: 21 × 21 × 4.5 cm Battery: 21 × 21 × 2.8 cm	Device with incorporated battery: 18 × 5.8 × 19 cm

Modified from Benson L. Semin Oncol Nurs. 2018;34:137–50 [78]*, TTFields* Tumor Treating Fields

### Skin Care

A meta-analysis has examined the safety of TTFields therapy delivered to the torso as reported in four pilot clinical studies [77]. In total, 192 patients were included in the analysis: 80 from STELLAR [33], 40 from the phase 1/2 PANOVA study in advanced pancreatic adenocarcinoma (EF- 20; NCT01971281) [38], 31 from the phase 1/2 INNOVATE study in recurrent ovarian carcinoma (EF- 22; NCT02244502) [87] and 41 from the phase 1/2 EF- 15 study (NCT00749346 l) in advanced non-small cell lung cancer [88]. TTFields therapy was administered concomitant with standard of care systemic chemotherapy in all studies. The only adverse event related to the medical device was the occurrence of dermatological adverse events in 58% of patients; grade 1–2 dermatitis was reported in 53% and grade 3–4 dermatological adverse events in 6% of patients. Grade 1–2 pruritus was reported in 9% of patients [77]. This meta-analysis further supported that the application of TTFields therapy to the torso was well tolerated, and had no incremental systemic side effects. More recently, similar safety findings were reported by the pivotal phase 3 LUNAR study of TTFields therapy applied to the thorax with a PD-(L)1 inhibitor or docetaxel in 133 patients with metastatic NSCLC. The most frequent TTFields-related adverse events were low grade skin irritation, with only 6% of patients reporting a grade 3 adverse event related to TTFields therapy, no deaths attributed to TTFields therapy, and no exacerbation of adverse events related to systemic therapy [36].

**Fig. 4:**
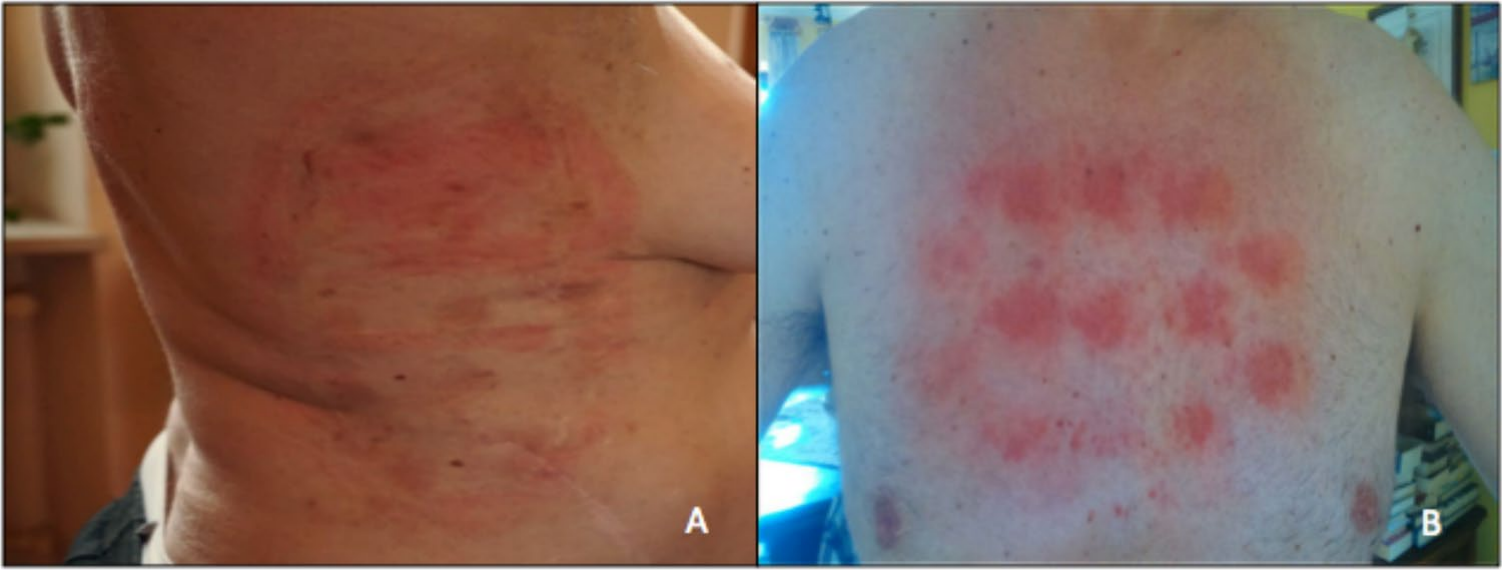
Skin adverse events with Tumor Treating Fields are mostly mild and related to contact with array components. It may be associated with (**A**) the adhesive tape and/or (**B**) the hydrogel-covered ceramic discs, Images are used with the permission of patients.

Dermatologic adverse events are principally related to contact between the skin and array components (such as the adhesive tape or hydrogel-covered ceramic discs), rather than the electric fields themselves (Fig. 4) [89, 90]. The major dermatologic adverse events can be classified as contact dermatitis (allergic or irritant), pruritus, hyperhidrosis, skin erosions, and infection [89–92]. In addition, pressure necrosis can develop due to shear forces during array replacement, and/or rubbing between the thorax and the device as a result of body movement, or mechanical pressure from the device hardware [90]. The main risk factors for dermatological adverse events are thought to be age, array contact with surgical scars, a history of contact dermatitis, excessive sweating, previous skin exposure to ultra violet or ionizing radiation, and recent administration of high-dose systemic corticosteroids [89, 90]. Skin moisturization is also a factor, as dehydrated skin is thinner, less elastic, and more susceptible to develop an adverse event than hydrated skin, which also typically heals more readily [90, 93].

Dermatological adverse events should be prevented or managed promptly to ensure consistent use of TTFields therapy and to optimize the clinical benefit [90]. Based on experience in clinical trials [30–33, 38, 87, 88], as well as post-marketing surveillance and expert consensus in patients with glioblastoma [94, 95], interventions should be based on patient and caregiver education, careful skin preparation and shaving, infection prevention, and array relocation [89, 90]. The use of heavy clothing should be minimized and/or clothes arranged to provide adequate ventilation, to reduce the effects of increased temperature, moisture, and occlusion [90]. Friction or pressure from the device itself can be mitigated using adhesive padding, such as silicone gel or a hydrocolloid dressing, whilst ensuring that array adhesion to the skin is unaffected [90]. The arrays should be changed at least twice a week, or even more frequently in some situations such as warmer weather, sweating, or after intense physical activity. They should be removed carefully and slowly using water (eg, under a shower) or using baby oil on the edge to enable removal of residual adhesive. The skin should be cleaned with water and hypoallergenic soap, and moisturized regularly with mild products that are compatible with TTFields therapy. During an array change, the skin should be inspected in order to promptly identify or monitor any skin AEs. Array position should be shifted by 1–2 cm at each change [89, 90].

Prophylaxis for skin damage is recommended to reduce the likelihood a patient develops TTFields therapy-related skin adverse events. It should begin as early as possible because skin adverse events may develop during the first month of therapy [90]. Simple routine skin care techniques (avoiding petroleum-based products that impede TTFields therapy) can be supplemented by topical steroids or calcineurin inhibitors [90]. In case of any sign of dermatitis, early treatment with a high potency topical corticosteroid, calcineurin inhibitor or phosphodiesterase 4 inhibitor is recommended [89, 90]. In addition, topical or oral antibiotics, and isolation of affected skin areas from adhesives and pressure could be indicated [89, 90]. Early management is particularly important in the thorax than other locations to prevent less severe adverse events from evolving into skin erosions and pressure necrosis (associated with skin movement and the larger array sizes used on the thorax than on the scalp, and also differences in skin thickness between the thorax and other locations) [90].

### Treatment Guidance and Dosimetry

Multiple parameters affect the distribution of TTFields therapy within the target region, including the (a) geometry and (b) dielectric properties of the skin, treated organ and surrounding tissues, as well as the distance between arrays [96–98]. TTFields do not attenuate in correlation to the distance from the array and may therefore be used for the treatment of deeply located tumors [96, 97]. Simulation studies have shown that meaningful TTFields field intensities of > 1 V/cm can easily be obtained throughout the lungs; moreover, the field distribution within the lungs can be tailored to deliver high field intensities to a specific region by adjusting the array position on the body. Therefore, personalized array layout planning is an integral part of TTFields therapy [99–101].

Preclinical studies in glioblastoma have shown that the anticancer effect of TTFields treatment is frequency and intensity dependent [45, 58]. Moreover, as discussed above, the efficacy of TTFields therapy is also time-dependent, with higher usage associated with improved patient survival [83, 84]. More recently, Ballo et al. performed a simulation-based analysis of 340 patients with newly diagnosed glioblastoma enrolled in the EF- 14 study [102]. They showed that PFS and OS correlated with the dose of TTFields delivered to the tumor bed. More specifically, they considered array layout, average duration of medical device use, and the average electrical current delivered to each patient as retrieved from patient records and log files of the TTFields device systems. Delivery of TTFields therapy to the patients was simulated and the spatial distribution of the fields analyzed. Local minimum dose density (LMiDD) was defined as the product of TTFields intensity, tissue-specific conductivities, and patient use [103]. The average LMiDD within a tumor bed comprising the gross tumor volume and a 3 mm-wide peritumoral boundary zone was calculated. Patients with average LMiDD in tumor bed ≥ 0.77 mW/cm^3^ had a significantly longer OS (25.2 vs 20.4 months of patients with LMiDD < 0.77 mW/cm^3^; HR 0.61 *p* = 0.003), as well as longer PFS (8.5 vs 6.7 months; HR 0.70, *p* = 0.02) [102].

These findings suggest that patient outcomes could be significantly improved with rigorous treatment planning, including using simulations to identify array layouts for optimal delivery of TTFields therapy to the tumor. In the future it may also be useful to adapt TTFields dosimetry to tumor size changes during treatment [104].

## Conclusion

TTFields therapy is a noninvasive, locoregional anticancer treatment, based on the delivery of electric fields by arrays applied to the patient skin covering and surrounding the tumor [29, 35]. The STELLAR study showed that TTFields therapy concomitant with standard chemotherapy (pemetrexed and either cisplatin or carboplatin) was an active and safe treatment for unresectable pleural mesothelioma [33]. No increase in systemic toxicities was observed when TTFields were applied to the torso, compared to standard chemotherapy alone. The only adverse event related to TTFields therapy was skin irritation underneath the arrays [33]. Based on these results, the NovoTTF- 100L system received FDA approval under the Humanitarian Device Exemption pathway and the Conformité Européenne mark in Europe [34, 105].

The decreased size and weight, combined with the newer ITE arrays of the second generation NovoTTF- 200 T system, improves its portability by unfit or frail patients, and generally causes minimal inconvenience to daily living. The systems are simple to use and designed to improve patient comfort, which potentially decreases the onset of skin reactions. At least one caregiver is needed who can help with array removal and accurate reapplication, and with operating the medical device. The daily usage of TTFields therapy, which can be negatively influenced by skin reactions [89, 90], is one of the key issues that may affect outcomes in patients with pleural mesothelioma, as already demonstrated in glioblastoma [83, 84]. Strategies for improving system use include patient and caregiver education and training, as well as proactive skin care [89, 90]. Finally, additional studies on TTFields therapy in pleural mesothelioma are needed, especially studies exploring the efficacy of TTFields therapy given concomitant with other therapeutic agents, including ICIs. The importance of this has been further highlighted recently by the LUNAR study in metastatic NSCLC finding that the survival benefit achieved with TTFields therapy was particularly pronounced when used with an ICI, compared to when used with docetaxel [36]. The ongoing TIGER Meso observational study (NCT05538806) aims to address some of these gaps by collecting real-world evidence (including efficacy, safety, feasibility and health-related QoL) of TTFields therapy in the routine clinical care setting [106].

Despite continuously increasing knowledge of its complex biology [107], and new treatments [11, 33], pleural mesothelioma remains incurable and has many unmet diagnostic and therapeutic needs [108]. There are many challenges to developing effective treatments, including a lack of a quick route to the use of precision targeted therapies, with one major issue being the inherent molecular and histological heterogeneity of the disease [109, 110].

Nevertheless, several milestones have been achieved, and new landmark studies suggest the direction of future treatments. ICIs [11, 24, 111, 112] will likely become the treatment backbone in the next years, although reliable biomarkers for patient selection and stratification are lacking so far [113, 114]. Interestingly, STELLAR found that patients with pleural mesothelioma of an epithelioid histology receiving TTFields with pemetrexed had longer PFS and OS than patients with non-epithelioid disease [33]. A similar differential response has been noted in other studies, including IND.227 and CheckMate- 743, although both trials showed a statistically significant benefit for ICIs as compared with chemotherapy alone in patients with non-epithelioid histology [11, 112]. Patient selection will be of utmost importance, with several studies dissecting the pleural mesothelioma tumor microenvironment showing that this disease should be considered as a series of heterogeneous entities with distinct immune cell infiltrates and immune checkpoint gene expression profiles [109, 115–117]. In addition, the development of personalized therapies requires further definition of the genomic landscape of pleural mesothelioma. Of note, the pivotal phase 3 LUNAR study identified a clinically significant improvement in OS in patients with metastatic NSCLC who received TTFields therapy concomitant with a PD-(L)1 inhibitor compared with those receiving PD-(L)1 inhibitor alone (18.5 months vs 10.8 months, HR 0.63 [0.41–0.96]; p = 0.03) [36]. Efficacy of the TTFields therapy plus PD-(L)1 inhibitor combination in metastatic NSCLC will be further assessed in the first-line setting (LUNAR- 2; NCT06216301) [118] and in the PD-(L)1 inhibitor re-challenge setting (LUNAR- 4; NCT06558799) [119]. The potential for future clinical first-line use of TTFields therapy in pleural mesothelioma remains to be elucidated; the ongoing TIGER Meso non-interventional study (NCT05538806) will provide further information on the use of TTFields therapy in routine clinical care of pleural mesothelioma, including data on its concomitant use with currently approved first-line systemic therapies such as nivolumab, ipilimumab, and pembrolizumab [106].

Unfortunately, unlike the many cancers driven by genetic alterations that activate growth-regulating kinases, pleural mesothelioma is mainly characterized by loss of tumor suppressor genes – and approaches that target these mechanisms remain elusive [120, 121]. Despite this, a few trials in selected pleural mesothelioma populations have been published [122]. Interestingly, extensive genomic analysis of pleural mesothelioma samples has suggested that clonal architecture may modulate immune surveillance and resistance to ICIs [123–125].

Strategies to overcome treatment resistance in pleural mesothelioma patients will likely be based around combination immunotherapies, including alternative checkpoint inhibitors to those acting on the PD- 1/PD-L1 and CTLA- 4 axes [126], angiogenesis inhibitors [117, 127], and chemotherapy [128]. In addition to continued investigation of TTFields therapy given concomitantly with immunotherapies, exploration into the addition of TTFields therapy to bevacizumab is also warranted; insights into the efficacy of these therapeutic combinations in pleural mesothelioma may be obtained from the ongoing TIGER Meso real-world study [129]. It is also likely that chemotherapy itself will continue to have a role in specific patient populations and settings [27, 113, 130, 131]. Antibody–drug conjugates targeting mesothelin, a cell surface glycoprotein commonly overexpressed by mesothelioma, have also been evaluated [132]. Although anetumab ravtansine failed to show superiority to vinorelbine in mesothelioma patients progressing on first-line pemetrexed/platinum chemotherapy [133], other newer antibody–drug conjugates have shown early promise as single agents or in combination with immune checkpoint inhibitors [134]. On the other hand, bi-specific antibodies targeting mesothelin, including bispecific T-cell engagers, are in early development [135, 136]. These surface-directed therapies could potentially be explored in combination with TTFields therapy, based on their mechanism of action and the non-overlapping toxicity profiles.

Within this landscape of evolving systemic treatments, TTFields therapy represents an additional option for pleural mesothelioma patients. The next steps concern how to optimize benefit from TTFields therapy. One of the most important is to ensure that TTFields therapy can be safely delivered with newer systemic agents, including immunotherapies, as they become introduced for pleural mesothelioma, without losing effectiveness. Additionally, whether the molecular characteristics of a tumor affect the efficacy of TTFields therapy remains unexplored in the clinical pleural mesothelioma setting [137]. Other than histology [43], it is currently unknown whether there are any specific biomarkers or mutations that predict response. We need more biological inquiry to plan the next generation of trials with TTFields therapy, possibly in selected populations [138]. Preclinical in vitro data and the availability of animal models [47] will certainly contribute to paving the way to the identification of predictive biomarkers of benefit.

There are also continuing efforts to improve the clinical delivery of TTFields therapy to tumors. Work is ongoing to define best practices for patient selection, patient and caregiver education, proactive skin care, and whether additional technological improvements can be leveraged for the device itself. Additionally, TTFields dosimetry and treatment planning is still in its early stages—many questions remain open [104]—and there is the potential for even better patient outcomes with further optimization. A rigorous numeric and reproducible definition of TTFields dose, and the realization of patient-specific computational models to mimic the distribution of TTFields and optimize array layouts, could contribute to definitively confirming the relationship between TTFields dose distribution and patient outcome. The routine application of treatment planning in the clinic will require processes and tools that accurately and quickly estimate field distributions. Moreover, adaptive tailoring of TTFields therapy to changes in the tumor might become possible via monitoring disease response/resistance using advanced imaging techniques, including radiomics [139, 140]. Overall, effective treatment planning will require the development of a quality assurance system similar to that used to plan radiation therapy. This could in turn lead to clinical guidelines regarding the accuracy of using computational modeling to determine array placement before starting treatment.

To conclude, TTFields therapy is a novel, emerging, and clinically versatile therapeutic tool that gives an additional treatment option in the battle against pleural mesothelioma without introducing additional toxicities other than mild-to-moderate skin irritation. Further gains over the next few years may occur through widespread adoption of accurate and quantitative treatment planning processes, and increased therapy usage driven by refinement of the medical device and optimized skin care regimens. Additionally, we may learn more about regimens that deliver TTFields therapy with other systemic therapies, and the molecular determinants of therapy efficacy in pleural mesothelioma.

## Key References

- Baas P, Scherpereel A, Nowak AK, et al. First-line nivolumab plus ipilimumab in unresectable malignant pleural mesothelioma (CheckMate 743): a multicentre, randomised, open-label, phase 3 trial. Lancet. 2021;397(10,272):375–386.
  - ⚬ This phase 3 trial is of major importance because it showed superiority of first-line immunotherapy (a combination of nivolumab and ipilimumab) over standard chemotherapy. This regimen was approved in 2020 by the FDA and EMA and was the first modification to first-line therapy for pleural mesothelioma in 15 years.
- Mun EJ, Babiker HM, Weinberg U, et al. Tumor-Treating Fields: A fourth modality in cancer treatment. Clin Cancer Res. 2018 Jan 15;24(2):266–275.
  - ⚬ This reference is of importance because it reviews and summarizes the mechanism of action for TTFields, how TTFields therapy is used by patients, and the clinical efficacy and safety of TTFields therapy in different clinical trials of various solid tumors.
- Ceresoli GL, Aerts JG, Dziadziuszko R, et al. Tumour Treating Fields in combination with pemetrexed and cisplatin or carboplatin as first-line treatment for unresectable malignant pleural mesothelioma (STELLAR): a multicentre, single-arm phase 2 trial. Lancet Oncol. 2019;20(12):1702–1709.
  - ⚬ This single-arm phase 2 study is of major importance because it showed a survival benefit for patients with unresectable malignant pleural mesothelioma treated with continuous TTFields and concomitant chemotherapy with pemetrexed and platinum agent. These results led to the approval of TTFields therapy in the US and Europe for the treatment of pleural mesothelioma.
- Leal T, Kotecha R, Ramlau R, et al. Tumor Treating Fields therapy with standard systemic therapy versus standard systemic therapy alone in metastatic non-small-cell lung cancer following progression on or after platinum-based therapy (LUNAR): a randomised, open-label, pivotal phase 3 study. Lancet Oncol. 2023;24:1002–1017.
  - ⚬ This phase 3 study is of major importance, demonstrating a significant increase in survival of patients with metastatic NSCLC that had progressed on platinum-based therapy treated with TTFields therapy concomitant with PD-(L)1 inhibitors or docetaxel. On the basis of these data, TTFields therapy was approved for the treatment of metastatic NSCLC in the US.
- Benson L. Tumor Treating Fields technology: alternating electric field therapy for the treatment of solid tumors. Semin Oncol Nurs. 2018 2018/05/01/;34(2):137–150.
  - ⚬ This reference is of outstanding importance because it provides guidance for oncology nurses on supporting and educating patients using TTFields therapy, which is crucial for maintaining adherence to treatment necessary for optimal survival benefits.
- Anadkat MJ, Lacouture M, Friedman A, et al. Expert guidance on prophylaxis and treatment of dermatologic adverse events with Tumor Treating Fields (TTFields) therapy in the thoracic region. Front Oncol. 2023 2023-January- 04;12:975,473.
  - ⚬ This reference is of major importance because it gives guidance for physicians on how to effectively minimize and treat skin related adverse effects from TTFields therapy, ensuring optimal treatment benefit.
- Ballo MT, Urman N, Lavy-Shahaf G, et al. Correlation of tumor treating fields dosimetry to survival outcomes in newly diagnosed glioblastoma: a large-scale numerical simulation-based analysis of data from the phase 3 EF- 14 randomized trial. Int J Radiat Oncol Biol Phys. 2019 Aug 1;104(5):1106–1113.
  - ⚬ This simulation-based study is of major importance as the first reported analysis describing patient-based dose responses to TTFields therapy and provides a rigorous definition for TTFields dose. This analysis demonstrated the clinically significant threshold of TTFields intensity as 1 V/cm.
- Bueno R, Stawiski EW, Goldstein LD, et al. Comprehensive genomic analysis of malignant pleural mesothelioma identifies recurrent mutations, gene fusions and splicing alterations. Nat Genet. 2016 Apr;48(4):407–16.
  - ⚬ These references are of importance because they describe the genetic landscape of pleural mesothelioma and highlight that the disease is highly heterogeneous and mainly characterized by loss of tumor suppressor genes, for which targeted approaches remain elusive.
- Hmeljak J, Sanchez-Vega F, Hoadley KA, et al. Integrative Molecular Characterization of Malignant Pleural Mesothelioma. Cancer Discov. 2018 Dec;8(12):1548–1565.
  - ⚬ These references are of importance because they describe the genetic landscape of pleural mesothelioma and highlight that the disease is highly heterogeneous and mainly characterized by loss of tumor suppressor genes, for which targeted approaches remain elusive.

## Data Availability

No datasets were generated or analysed during the current study.
